# miR-34a Regulates Multidrug Resistance via Positively Modulating OAZ2 Signaling in Colon Cancer Cells

**DOI:** 10.1155/2018/7498514

**Published:** 2018-08-02

**Authors:** Yong Li, Ping Gong, Ji-xue Hou, Wei Huang, Xiao-ping Ma, Yu-li Wang, Jing Li, Xiao-bin Cui, Na Li

**Affiliations:** ^1^Department of Radiology, Suining Central Hospital, Suining, Sichuan Province 629000, China; ^2^Department of Medical Oncology, First Affiliated Hospital of Medical College of Shihezi University, Shihezi, Xinjiang Uygur Autonomous Region 832000, China; ^3^Department of General Surgery, 2nd Division, First Affiliated Hospital of Medical College of Shihezi University, Shihezi, Xinjiang Uygur Autonomous Region 832000, China; ^4^Department of Pathology, First Affiliated Hospital of Medical College of Shihezi University, Shihezi, Xinjiang Uygur Autonomous Region 832000, China; ^5^Cancer Center of Suining Central Hospital, Suining, Sichuan Province 629000, China

## Abstract

Although aberrant expression of miR-34a, an essential tumor suppressor miRNA, has been frequently observed in colon cancer (CCa), whether miR-34a can regulate CCa progression by modulating other facets of this malignancy (such as multidrug resistance, MDR) remains unknown. Here, we report for the first time that miR-34a expression was significantly downregulated in clinical CCa samples from oxaliplatin-resistant patients and in experimentally established multidrug-resistant CCa cells. By using histoculture drug response assay, we further confirmed that clinical CCa samples with lower miR-34a expression appeared to be more resistant to chemotherapy. Functionally, ectopic expression of exogenous miR-34a resensitized multidrug-resistant HCT-8/OR cells to oxaliplatin treatment, whereas miR-34a inhibition augmented the oxaliplatin resistance in chemosensitive HCT-8 cells. Mechanistically, miR-34a positively regulated the mRNA stability of the ornithine decarboxylase antizyme 2 (OAZ2) by directly targeting its three prime untranslated region (3′UTR). Consequently, suppression of the expression of miR-34a/OAZ2 signaling by chemotherapeutic agents significantly enhanced the activation of MDR-associated ATP-binding cassette (ABC) transporters and antiapoptosis pathways, thus leading to MDR development in CCa cells. Collectively, our combined analysis reveals a critical role of miR-34a/OAZ2 cascade in conferring a proper cellular response to CCa chemotherapy.

## 1. Introduction

Colon cancer (CCa) is one of the most frequently diagnosed malignancies and one of the leading causes of cancer-related mortality worldwide [1]. Chemotherapy following surgery remains the main treatment for advanced CCa. Despite the advance in the understanding and application of chemotherapeutic drugs such as oxaliplatin and 5-fluorouracil, however, multidrug resistance (MDR), referring to mechanisms by which many cancers develop resistance to various chemotherapy drugs, occurs frequently and remains the major obstacle for CCa treatment [2]. The molecular mechanisms whereby MDR develops eventually are poorly understood. Evidence from clinical and basic studies suggests there are multiple factors involved: dysfunction of the efflux pump system [2]; deregulation of epigenetic modifications [3]; persistent activation of cytoprotective pathways [4, 5]; accumulation of excessive oxidative stress [6]; and abnormal activation of antiapoptosis signaling [7]. Apparently, the etiologies and contributing factors of MDR exist at different levels, and a proper integration of this complicated network is crucial for the pathogenesis.

MicroRNA (miRNA), a small noncoding RNA molecule binding to the three prime untranslated region (3′UTR) of its target gene mRNA, functions in posttranscriptional regulation of gene expression by affecting mRNA stability or repressing translation initiation [8]. A growing body of evidence suggests that deregulation of miRNA expression and function plays a key role in the development of drug resistance [9–11]. Of particular interest, miR-34a in CCa has attracted attention recently because it is controlled upstream by p53, a master transcriptional regulator that has been shown to fundamentally regulate cell apoptosis and tumorigenesis [12]. The expression of miR-34a is significantly repressed in CCa biopsies than in normal colon tissues [13]. Functionally, miR-34a regulates asymmetric stem cell division [14], inhibits tumorigenic epithelial-to-mesenchymal transition, and attenuates proliferation and metastasis in CCa [15]. Mechanistically, miR-34a acts as a tumor suppressor miRNA by regulating Notch [16], PDGFRA signaling, or multiple oncogenes [17]. Nevertheless, the functional involvement of miR-34a in other facets of CCa biology remains to be established.

Very recently, two research groups have reported independently that augmentation of miR-34a expression could resensitize CCa cells to 5-fluorouracil-based chemotherapy [18, 19], pointing to an essential involvement of this important tumor suppressor miRNA in the metabolism of chemotherapeutic agents. In this regard, the current study was designed to further investigate whether miR-34a plays a potential role in the pathogenesis of CCa MDR. Overall, our systematic analysis should pave the way for a better understanding of this important miRNA in CCa.

## 2. Materials and Methods

### 2.1. Human Samples

The human study is strictly conformed to the ethical standards of *Helsinki Declaration*, and the protocols were approved by the Ethics Committee of the First Affiliated Hospital of Medical College of Shihezi University. A total of 72 patients, who had received adjuvant oxaliplatin chemotherapy following the radical resection of histologically confirmed stage II (T2 and T3, N0, M0) or stage III (any T, N1 and 2, M0) CCa, were recruited from the Department of Medical Oncology in the First Affiliated Hospital of Medical College of Shihezi University during June 2014 and March 2017. Patients were subdivided into “primary” (complete response to oxaliplatin, *n* = 37) and “recurrent” (development of new signs of recurrent or metastatic lesions following surgery, *n* = 35) groups based on CT scan, examination of CEA levels, and colonoscopy [20]. In addition, adjacent normal colon tissues (*n* = 26) sampled at least 5 cm from primary tumors were obtained from chemotherapy-naive CCa patients and used as controls. Written informed consents were obtained from all patients.

### 2.2. Cell Treatment

The human colorectal adenocarcinoma cell line HCT-8, HCT-116, and SW-480 were obtained from American Type Culture Collection (ATCC, Manassas, VA, USA). The Genetic backgrounds of CCa cells used in the study have been listed in Supplementary Table 1. The oxaliplatin-resistant HCT-8, HCT-116, and SW-480 cells were established according to a previously reported protocol [7]. Initially, CCa cells were incubated with 4 *μ*M of oxaliplatin. When cells recovered to normal growth after a ~48 h incubation, cells were subcultured and continuingly treated with increased concentrations of oxaliplatin (1 *μ*M higher for each subculture). The cells were then subcultured for 11 times, and the final cells were resistant to 15 *μ*M of oxaliplatin and designated as CCa/OR. To manipulate the expression levels of miR-34a, cells were transfected with miR-34a mimic or miR-34a inhibitor, along with their corresponding negative controls, using HiPerFect Transfection Reagent (Qiagen, Shanghai, China). 48 h after transfection, cells were harvested and subjected to other assays. To overexpress the exogenous OAZ2, HCT-8 cells were transfected with pcDNA3.1^+^/DYK-OAZ2 or pcDNA3.1^+^ vector (GenScript, Nanjing, China) using HiPerFect Transfection Reagent, followed by selection with 300 *μ*g/mL G418 (InvivoGen, Hong Kong, China).

### 2.3. Cell Survival and Apoptosis

CCa cells (2.0 × 10^4^/well) were seeded in a 96-well plate. 24 h later, the culture medium was removed and cells were treated with a range of concentrations of oxaliplatin, paclitaxel, and vincristine as indicated for another 48 h. Subsequent cell viability was determined using the WST-1 viability assay (Roche Applied Science, Mannheim, Germany) according to the manufacturer's protocol. The optical density was measured at 450 nm with 650 nm as the reference wavelength with a microplate reader (xMark™ Microplate, Bio-Rad, Hercules, CA, USA). Based on the drug response curves, we determined the IC50 values (drug concentrations that achieved 50% growth inhibition) in respect to the different drug treatments using Bliss method [21]. Evaluation of cell apoptosis was carried out using an apoptosis ELISA kit (Roche Diagnostics, Shanghai, China), as per the manufacturer's instructions.

### 2.4. Histoculture Drug Response Assay (HDRA)

We took 19 patient samples and categorized them into “lower miR-34a level” group (with the relative miR-34a expression < 0.5, *n* = 8) and “higher miR-34a level” group (with the relative miR-34a expression > 1.5, *n* = 11), based on the RT-qPCR results as described below. Tumor samples were minced into small pieces (~0.5 mm in diameter) and placed on a collagen sponge gel in a 24-well plate. Samples were incubated with RPMI-1640 medium containing 20 *μ*g/mL cisplatin or 0.4 *μ*g/mL SN-38 (both from Sigma-Aldrich, Shanghai, China) for 7 days. After removal of culture medium, Hank's Balanced Salt Solution (Sigma-Aldrich) containing 5 mg/mL MTT (MedChemExpress, Shanghai, China) was added into each well, and the samples were incubated for another 8 h, followed by final spectrophotometry measured at 540 nm (Bio-Rad, Hercules, CA, USA). The inhibitory index was calculated according to a previous report [22]: the inhibitory index (%) = (1 − mean absorbance of treated tumor/g)/mean absorbance of untreated tumor/g.

### 2.5. Tumor Xenograft Model

*In vivo* chemosensitivity was determined using tumor xenograft model as described elsewhere [23]. Briefly, 0.1 mL of cell suspension (1 × 10^6^ cells/mL) was injected into the left flanks of male nude mice. When tumors grew to an average size of ~100 mm^3^, mice were injected subcutaneously with vehicle, 5 mg/kg/d of oxaliplatin every 2 days, for 28 d. Tumor volumes were recorded every 4 d after oxaliplatin administration and calculated using the formula: V = π/6x[(A + B)/2]^3^, using two perpendicular diameter measurements. All protocols involving animals, strictly conformed to the Guide for the Care and Use of Laboratory Animals, were approved by the local ACUC of the First Affiliated Hospital of Medical College of Shihezi University.

### 2.6. RT-qPCR

Total RNA was isolated from tissues samples and CCa cells using the Applied Biosystems™ MagMAX ™ Total RNA Isolation Kit (Thermo Fisher Scientific, Shanghai, China). Reverse transcription of miRNA was performed using miRNA-specific primer (3′-TGGCTCAGTTCAGCAGGAACAG-5′) by miScript II RT Kit (Qiagen). Reverse transcription of other gene targets were performed using High Capacity cDNA Reverse Transcription Kit (Thermo Fisher Scientific). Expression of miR-34a and other gene targets was then assessed using All-in-One™ miRNA RT-qPCR Reagent Kit (GeneCopoeia, Guangzhou, China), following the manufacturer's instructions. The primers used were: *18S*, 5′-CTCGCCGCGCTCTACCTACCTA-3′ and 5′-ATGAGCCATTCGCAGTTTCACTGTA-3′; OAZ2, 5′-GGTGAAGGTCTCTTCTTGGG-3′ and 5′-ACAATCCTTCTTTGCTCCCAT-3′. Parallel amplification of *18S* and U6 snRNA served as internal controls and the relative expression was determined using 2^–ΔΔCT^ method [24].

### 2.7. Immunoblotting

Western blotting or immunoblotting was carried out according to previous reports [25]. Membranes were probed with different primary antibodies as indicated in Supplementary Table 2. *β*-Actin served as a loading control. Immunostained bands were finally detected by using a ChemiDoc™ MP Imaging System (Bio-Rad).

### 2.8. Luciferase Reporter Assay

The genomic fragments harboring the putative miR-34a-binding sites in human OAZ2 3′UTR were subcloned into the pGL3-Luc reporter vector (Promega, Beijing, China) using Infusion 2.0 Dry-Down PCR cloning kit (Clontech, Shanghai, China). Promoter activity was further validated by mutation of the putative miR-34a-binding site on the OAZ2 3′UTR by replacing ACAC with CACA using the QuikChange II Site-Directed Mutagenesis Kit (Agilent, Santa Clara, CA, USA). For reporter assay, 0.5 *μ*g of pGL3-OAZ2 WT or pGL3-OAZ2 Mu, along with 0.05 *μ*g of pRL-SV40 plasmid and miR-34a mimic, was cotransfected into HeLa cells using FuGENE®6 (Promega). 48 h later, cells were harvested and subjected to luciferase activity measurements using the Promega Dual-Luciferase® Reporter Assay System.

### 2.9. Statistical Analysis

Statistical comparisons were performed using one-way ANOVA, followed by Tukey post hoc analyses. The relative correlation rate was determined using the Pearson chi-square test. Results are presented as mean ± S.E.M. and *P* < 0.05 was considered statistically significant.

## 3. Results

### 3.1. miR-34a Downregulation Is Associated with the Pathogenesis of Drug Resistance

**Figure 1:**
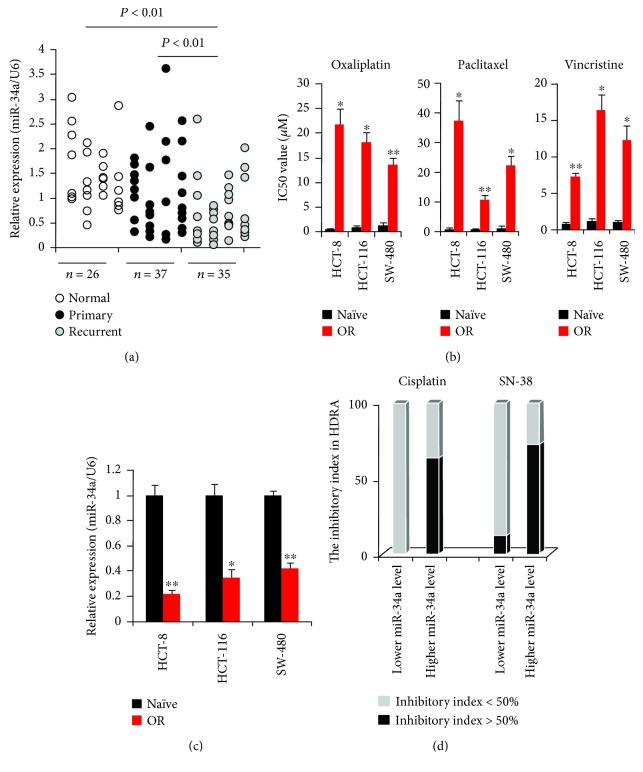
Downregulation of miR-34a expression is associated with multidrug resistance in colon cancer (CCa). (a) miR-34a expression in normal colon tissues, primary CCa tissues, and recurrent CCa tissues was determined using RT-qPCR. Fold change was determined for each sample relative to the internal control gene *18S*. Each value is a mean ± S.E.M. from three experiments. (b) CCa cells (2.0 × 10^4^/well) were seeded in a 96-well plate. 24 h later, cells were treated with different concentrations of oxaliplatin, paclitaxel, and vincristine as indicated, for another 48 h. Subsequent cell viability was determined using the WST-1 viability assay. The IC50 values (drug concentrations that achieved 50% growth inhibition) were determined as described in Materials and Methods. (c) Characterization of miR-34a expression in different CCa cells using RT-qPCR. (d) Histoculture drug response assay (HDRA) showing the differential chemosensitivities in clinical CCa tissues with different levels of miR-34a expression. ^∗^ *P* < 0.05 and ^∗∗^ *P* < 0.01 when compared to the Naive cells.

In the first attempt to explore the potential involvement of miR-34a in multidrug-resistant CCa, we evaluated the expression levels of miR-34a in CCa tissue samples from 72 patients, who had previously received adjuvant oxaliplatin chemotherapy. As shown by RT-qPCR (Figure 1(a)), miR-34a expression in oxaliplatin-resistant CCa (0.73 ± 0.38) was significantly decreased when compared to that in oxaliplatin-sensitive CCa (1.16 ± 0.79) and in adjacent normal colon tissues (1.49 ± 0.67). To validate this observation at the *in vitro* level, we established the oxaliplatin-resistant HCT-8/OR, HCT-116/OR, and SW-480/OR cells according to a previously validated protocol. Clearly, the HCT-8/OR, HCT-116/OR, and SW-480/OR cells were also more resistant to other chemotherapies including paclitaxel and vincristine (Figure 1(b)), warranting the use of these cells as the multidrug-resistant cell models in the current study. Interestingly, miR-34a expression in HCT-8/OR, HCT-116/OR, and SW-480/OR cells was significantly reduced compared with the parental HCT-8, HCT-116, and SW-480 cells (Figure 1(c)). We next tested whether miR-34a downregulation might correlate with drug resistance in clinical tumor samples. We took 19 patient samples and categorized them into “lower miR-34a level” group (with the relative miR-34a expression < 0.5, *n* = 8) and “higher miR-34a level” group (with the relative miR-34a expression > 1.5, *n* = 11), based on the RT-qPCR results. Given that cisplatin and SN-38 are frequently used for CCa chemotherapy [22], we subjected these samples to HDRA using these two drugs. In this assay, samples with an inhibitory index of >50% exhibit more sensitivity to anticancer agents [22]. The average inhibitory index (<50%) of tumors with lower miR-34a level treated with cisplatin and with SN-38 were 100% and 87.5%, respectively, whereas the average inhibitory index (<50%) of tumors with higher miR-34a level treated with cisplatin and with SN-38 were 36.4% and 27.3%, respectively (Figure 1(d)). Additionally, we overexpressed miR-34a in different colon cells by transfection of miR-34a mimic or Mimic NC. The cells with different transfections were then treated with 5 *μ*M cisplatin for 24 h, followed by apoptosis ELISA assay. As shown in Supplementary Figure 1, upregulation of miR-34a levels significantly enhanced cisplatin-induced cell death, comparing cisplatin + miR-34a mimic group to cisplatin + Mimic NC group or cisplatin group. These results are well consistent with our *in vivo* assay, stressing again that the counter-action of miR-34a against drug resistance appears to be universal in CCa samples.

### 3.2. Manipulation of miR-34a Expression Affects Chemosensitivity in CCa Cells

**Figure 2:**
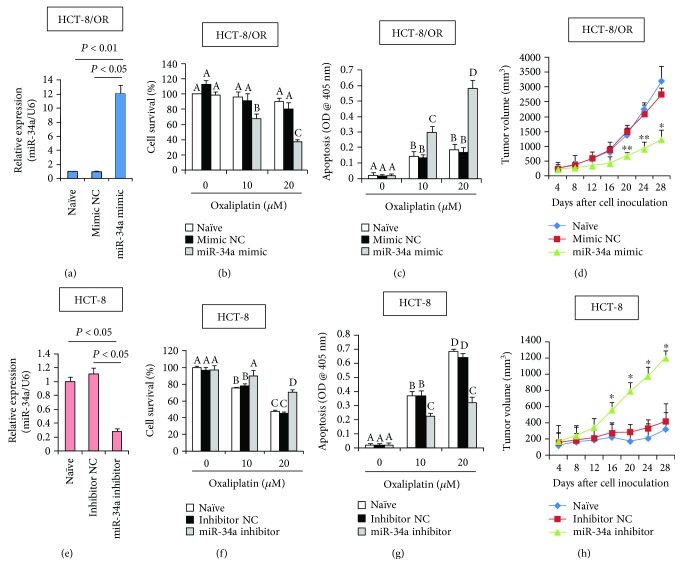
Manipulation of miR-34a expression affects chemosensitivity in CCa cells. (a) HCT-8/OR cells were transfected with miR-34a mimic or Mimic negative control (NC) as described in Materials and Methods. 48 h later, cells were collected, and the relative expression levels of miR-34a were assayed using RT-qPCR. HCT-8/OR cells with different transfections were incubated with different doses of oxaliplatin for 24 h, followed by cell viability assay (b) and apoptosis evaluation (c). Different superscript letters denote groups that are statistically different (*P* < 0.05). (d) HCT-8/OR cell-derived tumor xenograft model was established as described in Materials and Methods. Tumor volumes were measured every 4 days. ^∗^ *P* < 0.05 and ^∗∗^ *P* < 0.01 when comparing miR-34a mimic to Mimic NC. (e) HCT-8 cells were transfected with miR-34a inhibitor or Inhibitor NC as described in Materials and Methods. 48 h later, cells were collected and the relative expression levels of miR-34a were assayed using RT-qPCR. HCT-8 cells with different transfections were incubated with different doses of oxaliplatin for 24 h, followed by cell viability assay (f) and apoptosis evaluation (g). Different superscript letters denote groups that are statistically different (*P* < 0.05). (h) HCT-8 cell-derived tumor xenograft model was established as described in Materials and Methods. Tumor volumes were measured every 4 days. ^∗^ *P* < 0.05 and ^∗∗^ *P* < 0.01 when comparing miR-34a inhibitor to Inhibitor NC.

To ask directly whether miR-34a regulates drug resistance, we transfected the HCT-8/OR cells with miR-34a mimic and Mimic negative control (NC). A 48 h transfection resulted in a ~11.1-fold of induction in miR-34a expression level, as revealed by RT-qPCR analysis (Figure 2(a)). Interestingly, oxaliplatin-induced growth inhibition was notably amplified in HCT-8/OR/mimic cells compared to the mimic NC-transfected cells. This inhibitory effect appeared to be exerted in a dose-dependent manner (Figure 2(b)). Consistently, oxaliplatin-induced apoptosis was substantially induced in HCT-8/OR/mimic cells (Figure 2(c)). In agreement, the formation of xenograft tumors in oxaliplatin-challenged immunodeficient mice were significantly attenuated in HCT-8/OR cells by miR-34a overexpression (Figure 2(d)). Next, we investigated whether inhibition of endogenous miR-34a expression could influence the oxaliplatin chemosensitivity by transfecting the HCT-8 cells with miR-34a inhibitor and Inhibitor NC. The inhibition was verified by RT-qPCR analysis (Figure 2(e)). As expected, oxaliplatin-induced growth inhibition was significantly amplified in the HCT-8 cells depleted of endogenous miR-34a, which made the HCT-8 cells more resistant to oxaliplatin chemotherapy (Figure 2(f)). In accordance with this observation, oxaliplatin-induced apoptosis was effectively compromised upon miR-34a inhibition (Figure 2(g)), and the formation of xenograft tumors in oxaliplatin-challenged immunodeficient mice were also significantly enhanced in HCT-8 cells by miR-34a suppression (Figure 2(h)). We also confirmed the tumor suppressor effects of miR-34a in another two CCa cells (namely HCT-116 and SW-480, Supplementary Figure 2 and Supplementary Figure 3) Together, these data suggest that downregulation of miR-34a suppression may play a causative role during the pathogenesis of CCa chemoresistance.

### 3.3. miR-34a Inhibits Expressions of MRP2, P-gp, BCRP, and Bcl-2

**Figure 3:**
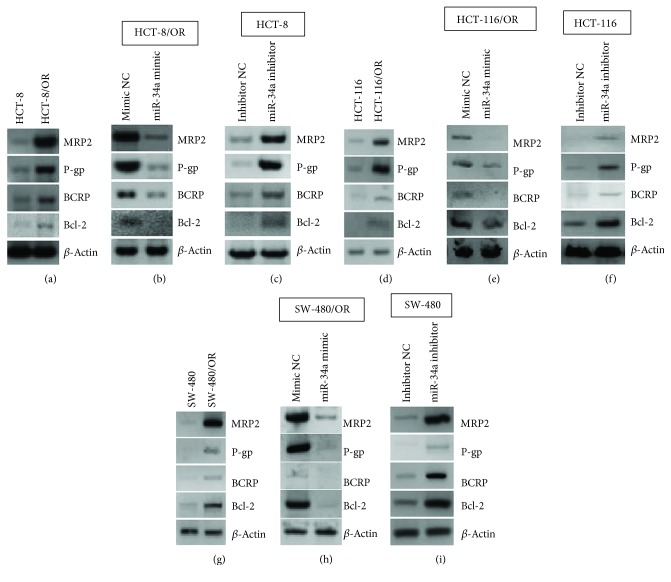
miR-34a inhibits the expression levels of signaling pathways associated with multidrug resistance. (a) Immunoblotting analysis of the expression levels of MRP2, P-gp, BCRP, and Bcl-2 in HCT-8 and HCT-8/OR cells. *β*-Actin served as loading control. (b) 48 h after transfection with miR-34a mimic or Mimic NC, HCT-8/OR cells were subjected to immunoblotting analysis of the expression levels of MRP2, P-gp, BCRP, and Bcl-2. (c) 48 h after transfection with miR-34a inhibitor or Inhibitor NC, HCT-8 cells were subjected to immunoblotting analysis of the expression levels of MRP2, P-gp, BCRP, and Bcl-2. (d) Immunoblotting analysis of the expression levels of MRP2, P-gp, BCRP, and Bcl-2 in HCT-116 and HCT-116/OR cells. (e) 48 h after transfection with miR-34a mimic or Mimic NC, HCT-116/OR cells were subjected to immunoblotting analysis of the expression levels of MRP2, P-gp, BCRP, and Bcl-2. (f) 48 h after transfection with miR-34a inhibitor or Inhibitor NC, HCT-116 cells were subjected to immunoblotting analysis of the expression levels of MRP2, P-gp, BCRP, and Bcl-2. (g) Immunoblotting analysis of the expression levels of MRP2, P-gp, BCRP, and Bcl-2 in SW-480 and SW-480/OR cells. (h) 48 h after transfection with miR-34a mimic or Mimic NC, SW-480/OR cells were subjected to immunoblotting analysis of the expression levels of MRP2, P-gp, BCRP, and Bcl-2. (i) 48 h after transfection with miR-34a inhibitor or Inhibitor NC, SW-480 cells were subjected to immunoblotting analysis of the expression levels of MRP2, P-gp, BCRP, and Bcl-2.

Accumulated data evidence the involvement of several ATP-binding cassette (ABC) transporters (*e.g.*, MRP2, P-gp, and BCRP) and antiapoptosis genes (*e.g.*, Bcl-2) in the development and progression of CCa chemoresistance. The ABC transporters pump out excessive intracellular drugs, thus leading to a significant impairment of chemotherapeutic effects [21]. Overexpression Bcl-2 can directly protect CCa cells from chemotherapy-induced apoptosis [26]. In our study, the expression levels of MRP2, P-gp, BCRP, and Bcl-2 were observed to be more robust in HCT-8/OR, HCT-116/OR, and SW-480/OR cells than in HCT-8, HCT-116, and SW-480 cells (Figures 3(a), 3(d), and 3(g)). Moreover, in HCT-8/OR, HCT-116/OR, and SW-480/OR cells, transfection with miR-34a mimic consistently attenuated the expression levels of MRP2, P-gp, BCRP, and Bcl-2 (Figures 3(b), 3(e), and 3(h)), whereas transfection with miR-34a inhibitor in HCT-8, HCT-116, and SW-480 cells resulted in a unanimous reduction in the expression levels of MRP2, P-gp, BCRP, and Bcl-2 when compared to control cells (Figures 3(c), 3(f), and 3(i)). Thus, miR-34a could regulate chemosensitivity via modulating several essential signaling transductions for MDR in CCa cells.

### 3.4. miR-34a Directly Targets OAZ2 3′UTR

**Figure 4:**
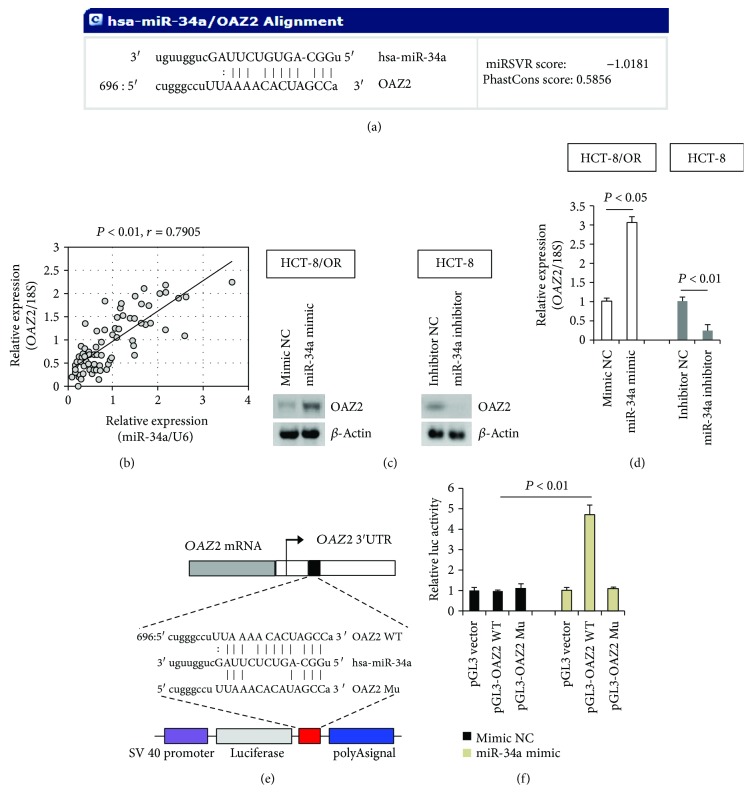
Direct regulation of OAZ2 expression by miR-34a. (a) The binding site of miR-34a in the *OAZ2* 3′UTR was analyzed using miRanda database. (b) Relative expression levels of miR-34a and *OAZ2* mRNA in clinical CCa samples were evaluated using RT-qPCR, followed by Pearson chi-square test. (c) Immunoblotting analysis of OAZ2 expression in HCT-8 and HCT-8/OR cells with different transfections. (d) RT-qPCR analysis of *OAZ2* mRNA expression in HCT-8 and HCT-8/OR cells with different transfections. (e) Simplified structure of the potential binding site of miR-34a onto the wild-type (WT) or mutant (Mu) *OAZ2* 3′UTR. (f) 0.5 *μ*g of pGL3-OAZ2 WT or pGL3-OAZ2 Mu, along with 0.05 *μ*g of pRL-SV40 plasmid and miR-34a mimic, was cotransfected into HeLa cells. 48 h later, cells were harvested and subjected to luciferase activity measurements.

Using combined analysis of public available human miRNA databases (miRanda and miRDB), we identified OAZ2 as a potential target of miR-34a (Figure 4(a)). In favor of this assumption, the *OAZ2* mRNA expression was found to be positively correlated to the expression level of miR-34a in clinical CCa samples (Figure 4(b)). Moreover, overexpression of the exogenous miR-34a in HCT-8/OR, HCT-116/OR, and SW-480/OR cells significantly upregulated OAZ2 expression, whereas inhibition of miR-34a expression by miR-34a inhibitor resulted in a dramatic decrease of OAZ2 expression in HCT-8, HCT-116, and SW-480 cells, at both translational (Figure 4(c), Supplementary Figure 4A and 4C) and transcriptional levels (Figure 4(d), Supplementary Figure 4B and 4D). These results suggest miR-34a may directly regulate the mRNA stability of OAZ2. To study whether miR-34a directly targets *OAZ2* 3′UTR, we performed luciferase reporter assays in HeLa cells using the fragments harboring the putative miR-34a-binding sites in *OAZ2* 3′UTR. Cotransfection with pGL3-OAZ2 WT and miR-34a mimic significantly increased the OAZ2 reporter activity relative to control vector. Furthermore, mutation of the binding site by replacing ACAC with CACA using the site-directed mutagenesis totally abolished miR-34a-elicited *OAZ2* 3′UTR activity (Figures 4(d) and 4(e)). Thus, miR-34a positively regulates *OAZ2* transcription by directly targeting its 3′UTR.

### 3.5. OAZ2 Overexpression Effectively Rescues the Chemosensitivity Impaired by miR-34a Deficiency

**Figure 5:**
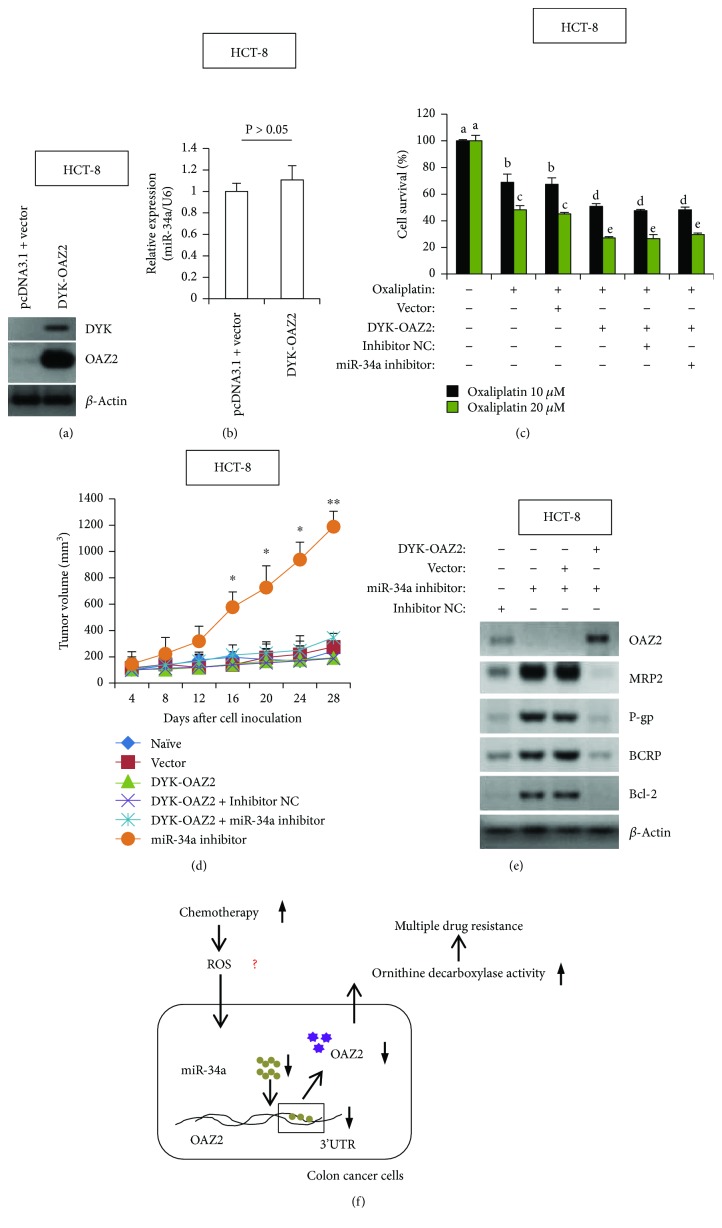
OAZ2 overexpression abolishes miR-34a deficiency-induced oxaliplatin-resistance. (a) HCT-8/OAZ2 cells were established as described in Materials and Methods. OAZ2 overexpression was confirmed by immunoblotting analysis. pcDNA3.1+/DYK vector is expressed in mammalian cells as a tagged protein with a C-terminal DYKDDDDK tag (DYKDDDDK is the same as FLAG® which is a registered trademark of Sigma-Aldrich). (b) RT-qPCR analysis of miR-34a expression in HCT-8/OAZ2 cells. (c) HCT-8/OAZ2 cells with different transfections were incubated with different doses of oxaliplatin for 24 h, followed by cell viability assay. Different superscript letters denote groups that are statistically different (*P* < 0.05). (d) HCT-8/OAZ2 cell-derived tumor xenografts model was established as described in Materials and Methods. Tumor volumes were measured every 4 days. ^∗^ *P* < 0.05 and ^∗∗^ *P* < 0.01 when comparing DYK-OAZ2 + miR-34a inhibitor to miR-34a inhibitor alone. (e) HCT-8/OAZ2 cells were transiently transfected with miR-34a inhibitor or Inhibitor NC for 48 h, followed by immunoblotting analysis of the expression levels of OAZ2, MRP2, P-gp, BCRP, and Bcl-2. (f) Proposed working model in the current study.

In the last experimental setting, we determined whether OAZ2 alone was sufficient to explain miR-34a deficiency-caused oxaliplatin resistance. HCT-8 cells were stably transfected with human OAZ2 cDNA clone, and OAZ2 overexpression was verified by immunoblotting (Figure 5(a)). Of note, upregulation of OAZ2 expression exerted no effects on miR-34a levels (Figure 5(b)), suggesting that miR-34a may act upstream of OAZ2 signaling. Incubation with different doses of oxaliplatin for 48 h resulted in a remarkable decrease of cell viability, whereas stable expression of OAZ2 caused a further reduction of cell viability in chemosensitive HCT-8 cells. Notably, cotransfection with miR-34a inhibitor in HCT-8/OAZ2 cells failed to reverse OAZ2-compromised cell viability (Figure 5(c)). In line with these *in vitro* data, OAZ2 overexpression potentiated the chemosensitivity of HCT-8/OAZ2 cells to oxaliplatin and maintained a relatively low formation of xenograft tumors in oxaliplatin-challenged immunodeficient mice, even in the presence of miR-34a inhibitor (Figure 5(d)). In agreement, augmentation of OAZ2 expression substantially abolished miR-34a inhibitor-elicited upregulation of MRP2, P-gp, BCRP, and Bcl-2, as evidenced by immunoblotting analysis (Figure 5(e)). Taken together, the available data confirm the relevance of the disruption of the miR-34a/OAZ2 signaling pathway in conferring CCa chemosensitivity.

## 4. Discussion

Despite the recent advances in the cancer treatment for advanced CCa, MDR that occurs through unidentified mechanisms remains the major challenge for chemotherapy. Accumulated data evidence a causative role of aberrant miRNA expression in the pathogenesis of chemoresistance [27]. To this end, the data presented here have provided novel insights into this issue by identifying miR-34a as an essential tumor suppressor miRNA during the pathogenesis of MDR in CCa. Treatment with chemotherapy drugs (*e.g.*, oxaliplatin, paclitaxel, and vincristine) inhibited miR-34a expression and accentuated the expression levels of several ABC transporters and antiapoptosis genes, thus leading to enhanced cell viability and decreased cell apoptosis in the presence of chemotherapy drugs, all of which are characteristic features of MDR. These results thus broaden our understanding of the epigenetic regulation of chemoresistance in CCa.

miR-34a was initially identified as a tumor suppressor miRNA, so its downregulation has been frequently observed in many malignancies including pancreatic cancer [28], squamous cell carcinoma [29], endometrial cancer [30], osteosarcoma [31], and hepatocellular carcinoma [32]. In line with these reports, we found here that miR-34a expression was significantly decreased in clinical samples from oxaliplatin-resistant CCa patients (Figure 1(a)), and in experimentally established multidrug-resistant CCa cells (Figure 1(c)). More importantly, we further confirmed that CCa tissues that express miR-34a at a lower level were more resistant to cisplatin- or SN-38-based chemotherapy, by using HDRA assay (Figure 1(d)). These data together indicate that deregulation of miR-34a is essentially involved in the development and progression of CCa, and an impaired expression of miR-34a may favor the pathogenesis of MDR in CCa. A recent study on the correlation between circulating miR-34a level and clinical parameters for CCa demonstrated that miR-34a expression was notably high in the plasma from CCa patients, and high levels of miR-34a expression could help to distinguish patients with advanced cancer from benign disease groups [33]. This is one possibility which may explain this discrepancy. By using high-throughput miRNA profiling, Pritchard et al. have observed that most of the solid tumor circulating miRNA biomarkers reported previously were highly expressed in one or more blood cell types, indicating that blood cells could be major contributors to a majority of circulating miRNAs [34]. In this context, it is very likely that miR-34a is also blood cell-expressed, and therefore, the high level of circulating miR-34a expression in the abovementioned study may not be the true reflection of miR-34a dynamics in CCa tissues. Nevertheless, several other studies [35–38], including our own, have verified that downregulation of miR-34a potentiates chemoresistance in CCa cells.

The mechanisms by which miR-34a expression is compromised in multidrug-resistant CCa remain to be revealed, but one factor has been so far reported to determine the level of miR-34a expression. Increasing data evidence that excessive production of reactive oxygen species (ROS) plays a pivotal role during the cancerous progression, and miRNAs have been confirmed to be functionally involved in the downstream signaling pathway of oxidative stress [39, 40]. To this end, by employing robust rank aggregation (RRA) assay, Wan *et al.* have demonstrated that miR-34a is one of several essential oxidative stress-responsive miRNAs during carcinogenesis [41]. Overproduction of ROS negatively modulates miR-34a expression under different pathological conditions including osteoarthritis [42], chronic obstructive pulmonary disease [43], diabetes [44], and cancer [45]. Given that chemotherapeutic drugs are usually DNA-damaging agents and they continuingly cause excessive oxidative stress by attenuating the expression levels of essential antioxidants, especially during the combination chemotherapy [46], we reason that it is the superfluous ROS that significantly impairs miR-34a expression during the pathogenesis of MDR. This hypothesis is currently being under investigation in our lab.

Another intriguing question addressed in the present communication is how miR-34a deficiency potentiates MDR in CCa cells. We have shown that miR-34a directly targeted OAZ2 3′UTR and positively regulated the expression levels of OAZ2 (Figure 4). More importantly, we have successfully rescued the chemosensitivity by expressing the exogenous OAZ2 in HCT-8 cells that had been deprived of endogenous miR-34a (Figure 5). Thus, OAZ2 serves as the main downstream effector of miR-34a signaling in CCa. Overexpression of ornithine decarboxylase (ODC) is a well-known pathological feature of chemoresistant cancers. ODC augmentation has been frequently observed to be involved in the pathogenesis of oxaliplatin resistance, paclitaxel resistance, vincristine resistance, and cisplatin resistance in various cancer types [47–50]. As an endogenous ODC-activity antagonist, the antizyme 2 (OAZ2) expression is expected to be downregulated during cancer progression. Indeed, a dramatic decrease in the expression of OAZ2 has been reported in gastric cancer [51] and neuroblastoma [52], and high levels of OAZ2 expression are positively correlated with increased survival rate and favorable clinical prognosis. In this context, we reason that the chemosensitivity-promoting effects of OAZ2 may exhibit through antagonizing ODC activity. However, a major unresolved question is how the expression of OAZ2 is transcriptionally or posttranscriptionally regulated along cancerous progression. We have shown here miR-34a could positively regulate the mRNA stability of OAZ2 by directly targeting its 3′UTR (Figure 4). In accordance with our observations, neurobiology studies have previously demonstrated that promoter methylation negatively regulates neuronal *OAZ2* transcription under certain pathological conditions [52]. Thus, the available data strongly suggest that epigenetic mechanisms (e.g., DNA methylation and miRNA regulation) play important roles in regulating OAZ2 expression. Future endeavor in this field should provide novel chemosensitizing strategies against drug resistance in CCa.

In conclusion, the data provided here have elucidated a critical role of miR-34a/OAZ2 cascade in conferring a proper cellular response to CCa chemotherapy. Inhibition of miR-34a by different chemotherapeutic agents attenuates the mRNA stability of OAZ2, reduces OAZ2 expression, and enhances the activation of several MDR-associated signaling pathways (Figure 5(f)). Consequently, by revealing how OAZ2 is regulated by miR-34a at the posttranscriptional level, our findings suggest that miR-34a mimic and OAZ2 agonist may be repositioned to suppress cancer progression, dampen tumor evolution, and decrease the probability of adverse outcomes by MDR in colon cancer.

## Data Availability

The data used to support the findings of this study are included within the article.

## Supplementary Materials

Supplementary 1 Supplementary Table 1: genetic backgrounds of colon cancer cells used in the study.

Supplementary 2 Supplementary Table 2: sources of antibodies and the working dilutions that were used in this study.

Supplementary 3 Supplementary Figure 1: upregulation of miR-34a expression promotes chemosensitivity in CCa cells. (A) HCT-8, HCT-116, and SW-480 cells were transfected with miR-34a mimic or Mimic negative control (NC) as described in Materials and Methods. 48 h later, cells were collected, and the relative expression levels of miR-34a were assayed using RT-qPCR. (B) CCa cells with different transfections were treated with 5 *μ*M cisplatin for 24 h, followed by apoptosis ELISA assay. Different superscript letters denote groups that are statistically different (*P* < 0.05).

Supplementary 4 Supplementary Figure 2: manipulation of miR-34a expression affects chemosensitivity in HCT-116 cells. (A) HCT-116/OR cells were transfected with miR-34a mimic or Mimic negative control (NC) as described in Materials and Methods. 48 h later, cells were collected, and the relative expression levels of miR-34a were assayed using RT-qPCR. HCT-116/OR cells with different transfections were incubated with different doses of oxaliplatin for 24 h, followed by cell viability assay (B) and apoptosis evaluation (C). Different superscript letters denote groups that are statistically different (*P* < 0.05). (D) HCT-116/OR cell-derived tumor xenograft model was established as described in Materials and Methods. Tumor volumes were measured every 4 days. ^∗^ *P* < 0.05 and ^∗∗^ *P* < 0.01 when comparing miR-34a mimic to Mimic NC. (E) HCT-116 cells were transfected with miR-34a inhibitor or Inhibitor NC as described in Materials and Methods. 48 h later, cells were collected, and the relative expression levels of miR-34a were assayed using RT-qPCR. HCT-116 cells with different transfections were incubated with different doses of oxaliplatin for 24 h, followed by cell viability assay (F) and apoptosis evaluation (G). Different superscript letters denote groups that are statistically different (*P* < 0.05). (H) HCT-116 cell-derived tumor xenograft model was established as described in Materials and Methods. Tumor volumes were measured every 4 days. ^∗^ *P* < 0.05 and ^∗∗^ *P* < 0.01 when comparing miR-34a inhibitor to Inhibitor NC.

Supplementary 5 Supplementary Figure 3: Manipulation of miR-34a expression affects chemosensitivity in SW-480 cells. (A) SW-480/OR cells were transfected with miR-34a mimic or Mimic negative control (NC) as described in Materials and Methods. 48 h later, cells were collected, and the relative expression levels of miR-34a were assayed using RT-qPCR. SW-480/OR cells with different transfections were incubated with different doses of oxaliplatin for 24 h, followed by cell viability assay (B) and apoptosis evaluation (C). Different superscript letters denote groups that are statistically different (*P* < 0.05). (D) SW-480/OR cell-derived tumor xenograft model was established as described in Materials and Methods. Tumor volumes were measured every 4 days. ^∗^ *P* < 0.05 and ^∗∗^ *P* < 0.01 when comparing miR-34a mimic to Mimic NC. (E) SW-480 cells were transfected with miR-34a inhibitor or Inhibitor NC as described in Materials and Methods. 48 h later, cells were collected, and the relative expression levels of miR-34a were assayed using RT-qPCR. SW-480 cells with different transfections were incubated with different doses of oxaliplatin for 24 h, followed by cell viability assay (F) and apoptosis evaluation (G). Different superscript letters denote groups that are statistically different (*P* < 0.05). (H) SW-480 cell-derived tumor xenograft model was established as described in Materials and Methods. Tumor volumes were measured every 4 days. ^∗^ *P* < 0.05 and ^∗∗^ *P* < 0.01 when comparing miR-34a inhibitor to Inhibitor NC.

Supplementary 6 Supplementary Figure 4: manipulation of miR-34a expression affects OAZ2 expression levels in CCa cells. (A) Immunoblotting analysis of OAZ2 expression in HCT-116 and HCT-116/OR cells with different transfections. (B) RT-qPCR analysis of *OAZ2* mRNA expression in HCT-116 and HCT-116/OR cells with different transfections. (C) Immunoblotting analysis of OAZ2 expression in SW-480 and SW-480/OR cells with different transfections. (B) RT-qPCR analysis of *OAZ2* mRNA expression in SW-480 and SW-480/OR cells with different transfections.
