# Supplementary material for: miR-34a Regulates Multidrug Resistance via Positively Modulating OAZ2 Signaling in Colon Cancer Cells
Source: J Immunol Res. 2018 Aug 2;2018:7498514. doi: 10.1155/2018/7498514 (PMC6098920; doi:10.1155/2018/7498514)
Supplement: Supplementary 1 — Supplementary Table 1: genetic backgrounds of colon cancer cells used in the study. [file 7498514.f1.doc]

**Supplementary Table 1** Genetic backgrounds of colon cancer cells used in the study

| Cell line | Disease | Genes expressed |
| --- | --- | --- |
| HCT-8 | Colorectal adenocarcinoma | CEA, alkaline phosphatase; keratin |
| HCT-116 | Colorectal carcinoma | CEA |
| SW-480 | Colorectal adenocarcinoma | TGF-β, myc +; myb + ; ras +; fos +; sis +; p53 +; abl-; ros -; src -, HLA A2, B8, B17; blood type A; Rh+ |
