# Supplementary material for: miR-34a Regulates Multidrug Resistance via Positively Modulating OAZ2 Signaling in Colon Cancer Cells
Source: J Immunol Res. 2018 Aug 2;2018:7498514. doi: 10.1155/2018/7498514 (PMC6098920; doi:10.1155/2018/7498514)
Supplement: Supplementary 2 — Supplementary Table 2: sources of antibodies and the working dilutions that were used in this study. [file 7498514.f2.doc]

**Supplementary Table 2** Sources of antibodies and the working dilutions that were used in this study

| Antibody | Vendor | Catalog no. | Dilutions |
| --- | --- | --- | --- |
| Rabbit anti-MRP2 | Abcam, (Shanghai, China) | ab187644 | 1:1000 |
| Rabbit anti-P-gp | Abcam, (Shanghai, China) | ab129450 | 1:1000 |
| Rabbit anti-BCRP | Proteintech (Rosemont, IL, USA) | 10051-1-AP | 1:1000 |
| Rabbit anti-Bcl-2 | Cell Signaling Technology, Inc. (Shanghai, China) | #2872 | 1:1500 |
| Rabbit anti-OAZ2 | Abcam, (Shanghai, China) | ab108000 | 1:1000 |
| Rabbit anti-β-ACTIN | Cell Signaling Technology, Inc. (Shanghai, China) | #4967 | 1:2500 |
| Goat anti-Rabbit IgG -HRP secondary antibody | Abcam, (Shanghai, China) | ab97200 | 1:10000 |
