# Supplementary material for: miR-34a Regulates Multidrug Resistance via Positively Modulating OAZ2 Signaling in Colon Cancer Cells
Source: J Immunol Res. 2018 Aug 2;2018:7498514. doi: 10.1155/2018/7498514 (PMC6098920; doi:10.1155/2018/7498514)
Supplement: Supplementary 3 — Supplementary Figure 1: upregulation of miR-34a expression promotes chemosensitivity in CCa cells. (A) HCT-8, HCT-116, and SW-480 cells were transfected with miR-34a mimic or Mimic negative control (NC) as described in Materials and Methods. 48 h later, cells were collected, and the relative expression levels of miR-34a were assayed using RT-qPCR. (B) CCa cells with different transfections were treated with 5 μM cisplatin for 24 h, followed by apoptosis ELISA assay. Different superscript letters denote groups that are statistically different (P < 0.05). [file 7498514.f3.pptx]

## Slide 1
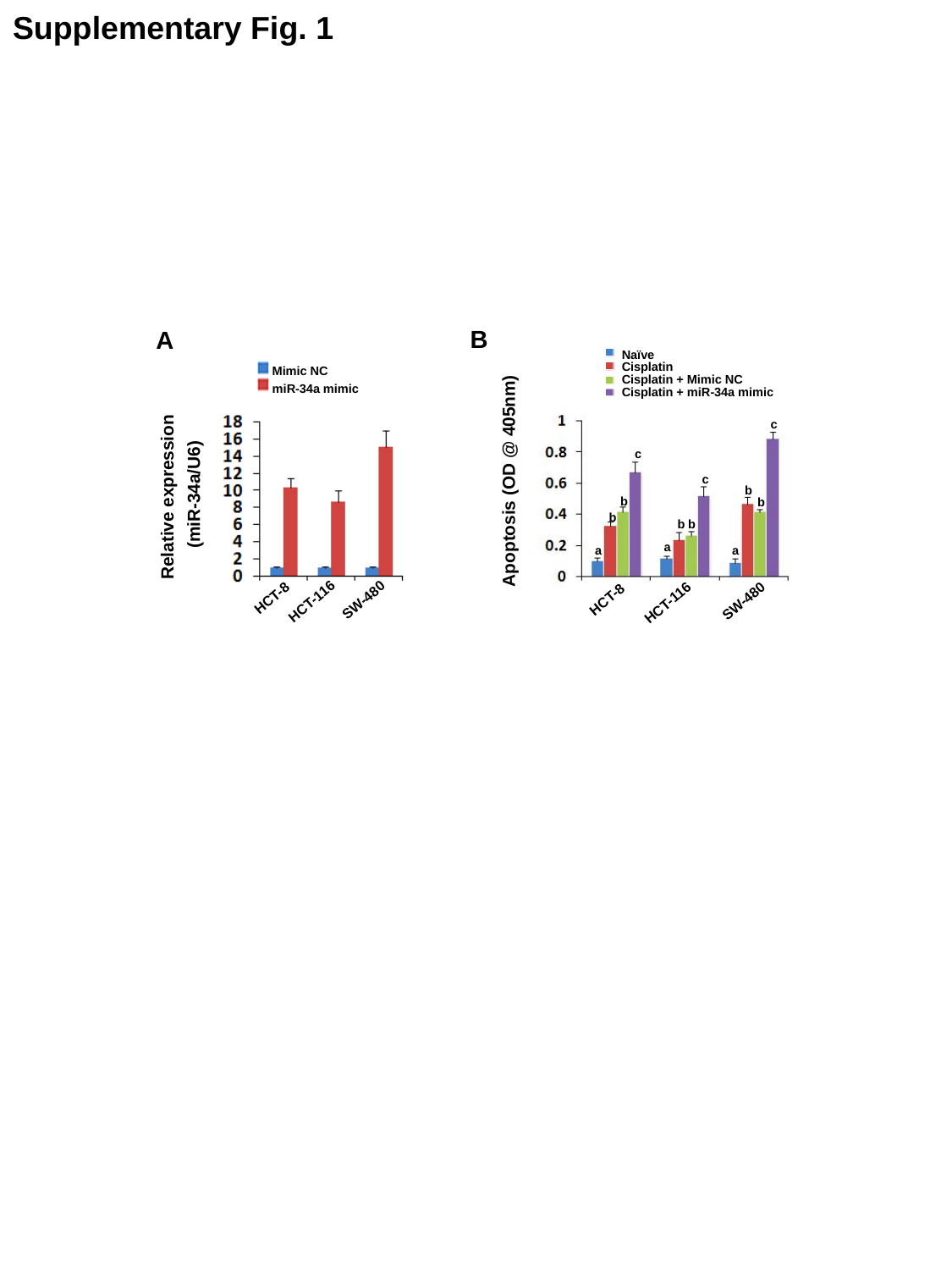

Supplementary Fig. 1
B
A
Naïve
Cisplatin
Cisplatin + Mimic NC
Cisplatin + miR-34a mimic
Mimic NC
miR-34a mimic
c
c
Apoptosis (OD @ 405nm)
Relative expression
(miR-34a/U6)
c
b
b
b
b
b
b
a
a
a
HCT-116
SW-480
HCT-116
SW-480
HCT-8
HCT-8
