# Supplementary material for: miR-34a Regulates Multidrug Resistance via Positively Modulating OAZ2 Signaling in Colon Cancer Cells
Source: J Immunol Res. 2018 Aug 2;2018:7498514. doi: 10.1155/2018/7498514 (PMC6098920; doi:10.1155/2018/7498514)
Supplement: Supplementary 4 — Supplementary Figure 2: manipulation of miR-34a expression affects chemosensitivity in HCT-116 cells. (A) HCT-116/OR cells were transfected with miR-34a mimic or Mimic negative control (NC) as described in Materials and Methods. 48 h later, cells were collected, and the relative expression levels of miR-34a were assayed using RT-qPCR. HCT-116/OR cells with different transfections were incubated with different doses of oxaliplatin for 24 h, followed by cell viability assay (B) and apoptosis evaluation (C). Different superscript letters denote groups that are statistically different (P < 0.05). (D) HCT-116/OR cell-derived tumor xenograft model was established as described in Materials and Methods. Tumor volumes were measured every 4 days. ∗ P < 0.05 and ∗∗ P < 0.01 when comparing miR-34a mimic to Mimic NC. (E) HCT-116 cells were transfected with miR-34a inhibitor or Inhibitor NC as described in Materials and Methods. 48 h later, cells were collected, and the relative expression levels of miR-34a were assayed using RT-qPCR. HCT-116 cells with different transfections were incubated with different doses of oxaliplatin for 24 h, followed by cell viability assay (F) and apoptosis evaluation (G). Different superscript letters denote groups that are statistically different (P < 0.05). (H) HCT-116 cell-derived tumor xenograft model was established as described in Materials and Methods. Tumor volumes were measured every 4 days. ∗ P < 0.05 and ∗∗ P < 0.01 when comparing miR-34a inhibitor to Inhibitor NC. [file 7498514.f4.pptx]

## Slide 1
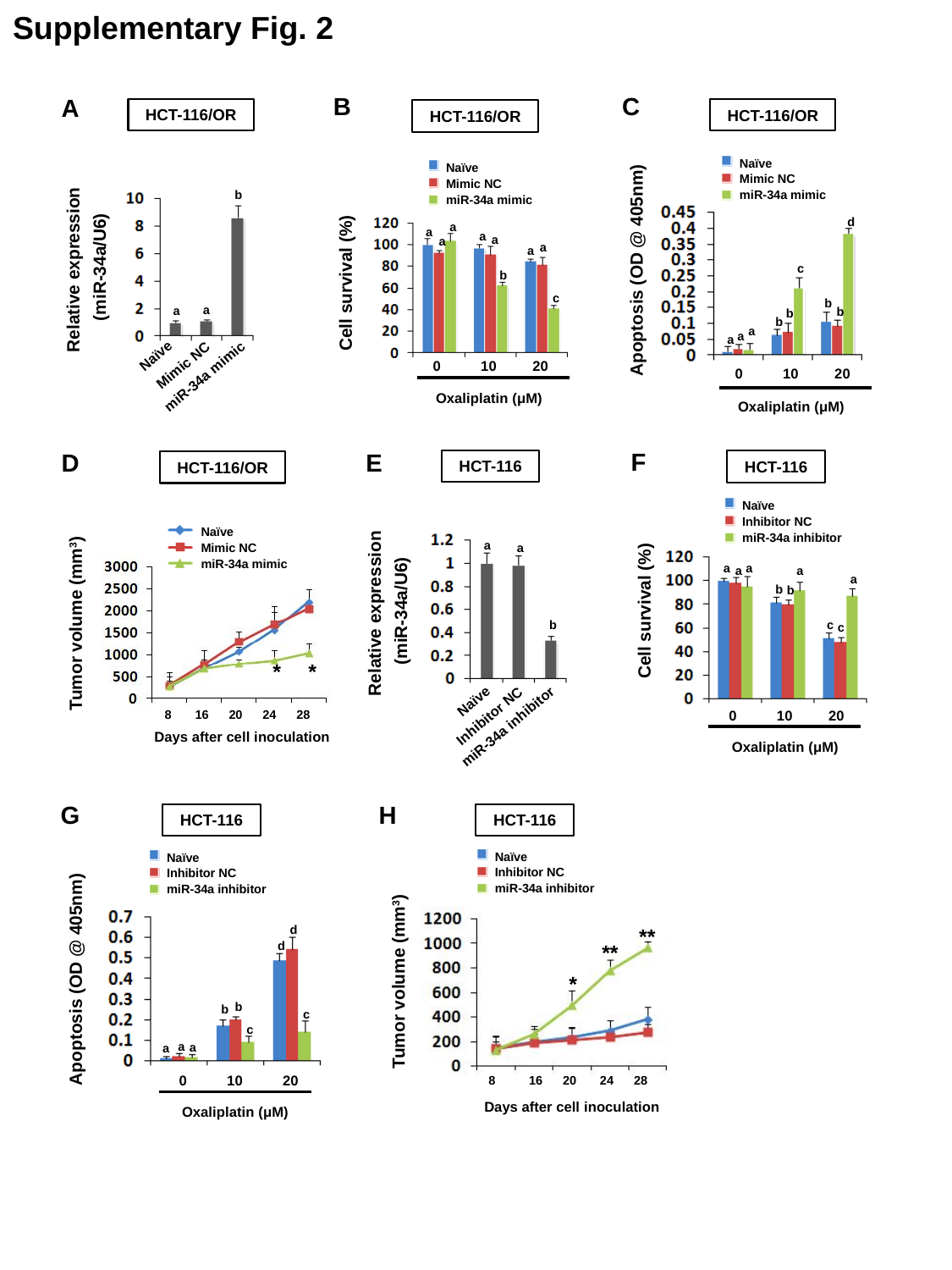

Supplementary Fig. 2
B
C
A
HCT-116/OR
HCT-116/OR
HCT-116/OR
Naïve
Mimic NC
miR-34a mimic
Naïve
Mimic NC
miR-34a mimic
b
d
a
a
a
a
a
a
Relative expression
(miR-34a/U6)
a
Apoptosis (OD @ 405nm)
c
b
Cell survival (%)
c
b
a
a
b
b
b
a
a
a
Naïve
0 10 20
0 10 20
Mimic NC
miR-34a mimic
Oxaliplatin (μM)
Oxaliplatin (μM)
F
E
D
HCT-116
HCT-116
HCT-116/OR
Naïve
Inhibitor NC
miR-34a inhibitor
Naïve
Mimic NC
miR-34a mimic
a
a
a
a
a
a
a
b
b
Relative expression
(miR-34a/U6)
Cell survival (%)
Tumor volume (mm3)
c
b
c
*
*
Naïve
0 10 20
8 16 20 24 28
Inhibitor NC
miR-34a inhibitor
Days after cell inoculation
Oxaliplatin (μM)
G
H
HCT-116
HCT-116
Naïve
Inhibitor NC
miR-34a inhibitor
Naïve
Inhibitor NC
miR-34a inhibitor
d
**
d
**
Apoptosis (OD @ 405nm)
Tumor volume (mm3)
*
b
b
c
c
a
a
a
0 10 20
8 16 20 24 28
Days after cell inoculation
Oxaliplatin (μM)
