# Supplementary material for: miR-34a Regulates Multidrug Resistance via Positively Modulating OAZ2 Signaling in Colon Cancer Cells
Source: J Immunol Res. 2018 Aug 2;2018:7498514. doi: 10.1155/2018/7498514 (PMC6098920; doi:10.1155/2018/7498514)
Supplement: Supplementary 5 — Supplementary Figure 3: Manipulation of miR-34a expression affects chemosensitivity in SW-480 cells. (A) SW-480/OR cells were transfected with miR-34a mimic or Mimic negative control (NC) as described in Materials and Methods. 48 h later, cells were collected, and the relative expression levels of miR-34a were assayed using RT-qPCR. SW-480/OR cells with different transfections were incubated with different doses of oxaliplatin for 24 h, followed by cell viability assay (B) and apoptosis evaluation (C). Different superscript letters denote groups that are statistically different (P < 0.05). (D) SW-480/OR cell-derived tumor xenograft model was established as described in Materials and Methods. Tumor volumes were measured every 4 days. ∗ P < 0.05 and ∗∗ P < 0.01 when comparing miR-34a mimic to Mimic NC. (E) SW-480 cells were transfected with miR-34a inhibitor or Inhibitor NC as described in Materials and Methods. 48 h later, cells were collected, and the relative expression levels of miR-34a were assayed using RT-qPCR. SW-480 cells with different transfections were incubated with different doses of oxaliplatin for 24 h, followed by cell viability assay (F) and apoptosis evaluation (G). Different superscript letters denote groups that are statistically different (P < 0.05). (H) SW-480 cell-derived tumor xenograft model was established as described in Materials and Methods. Tumor volumes were measured every 4 days. ∗ P < 0.05 and ∗∗ P < 0.01 when comparing miR-34a inhibitor to Inhibitor NC. [file 7498514.f5.pptx]

## Slide 1
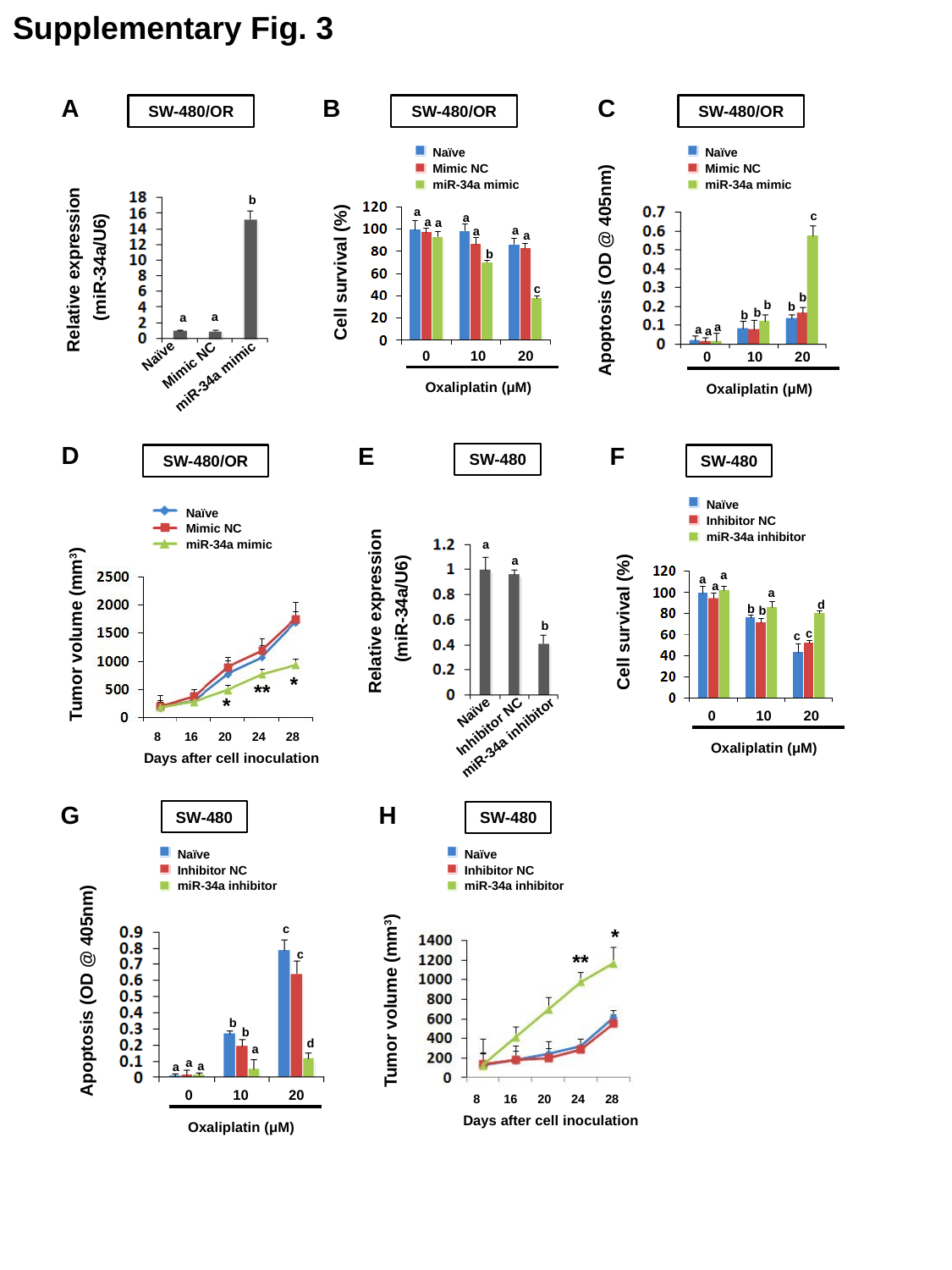

Supplementary Fig. 3
B
C
A
SW-480/OR
SW-480/OR
SW-480/OR
Naïve
Mimic NC
miR-34a mimic
Naïve
Mimic NC
miR-34a mimic
b
a
c
a
a
a
a
a
a
Relative expression
(miR-34a/U6)
b
Apoptosis (OD @ 405nm)
Cell survival (%)
c
b
b
b
b
b
a
a
a
a
a
0 10 20
0 10 20
Naïve
Mimic NC
miR-34a mimic
Oxaliplatin (μM)
Oxaliplatin (μM)
D
E
F
SW-480
SW-480/OR
SW-480
Naïve
Inhibitor NC
miR-34a inhibitor
Naïve
Mimic NC
miR-34a mimic
a
a
a
a
a
Relative expression
(miR-34a/U6)
a
d
b
b
Cell survival (%)
b
Tumor volume (mm3)
c
c
*
**
*
0 10 20
Naïve
Inhibitor NC
8 16 20 24 28
miR-34a inhibitor
Oxaliplatin (μM)
Days after cell inoculation
G
H
SW-480
SW-480
Naïve
Inhibitor NC
miR-34a inhibitor
Naïve
Inhibitor NC
miR-34a inhibitor
c
*
c
**
Apoptosis (OD @ 405nm)
Tumor volume (mm3)
b
b
d
a
a
a
a
0 10 20
8 16 20 24 28
Days after cell inoculation
Oxaliplatin (μM)
