# Supplementary material for: miR-34a Regulates Multidrug Resistance via Positively Modulating OAZ2 Signaling in Colon Cancer Cells
Source: J Immunol Res. 2018 Aug 2;2018:7498514. doi: 10.1155/2018/7498514 (PMC6098920; doi:10.1155/2018/7498514)
Supplement: Supplementary 6 — Supplementary Figure 4: manipulation of miR-34a expression affects OAZ2 expression levels in CCa cells. (A) Immunoblotting analysis of OAZ2 expression in HCT-116 and HCT-116/OR cells with different transfections. (B) RT-qPCR analysis of OAZ2 mRNA expression in HCT-116 and HCT-116/OR cells with different transfections. (C) Immunoblotting analysis of OAZ2 expression in SW-480 and SW-480/OR cells with different transfections. (B) RT-qPCR analysis of OAZ2 mRNA expression in SW-480 and SW-480/OR cells with different transfections. [file 7498514.f6.pptx]

## Slide 1
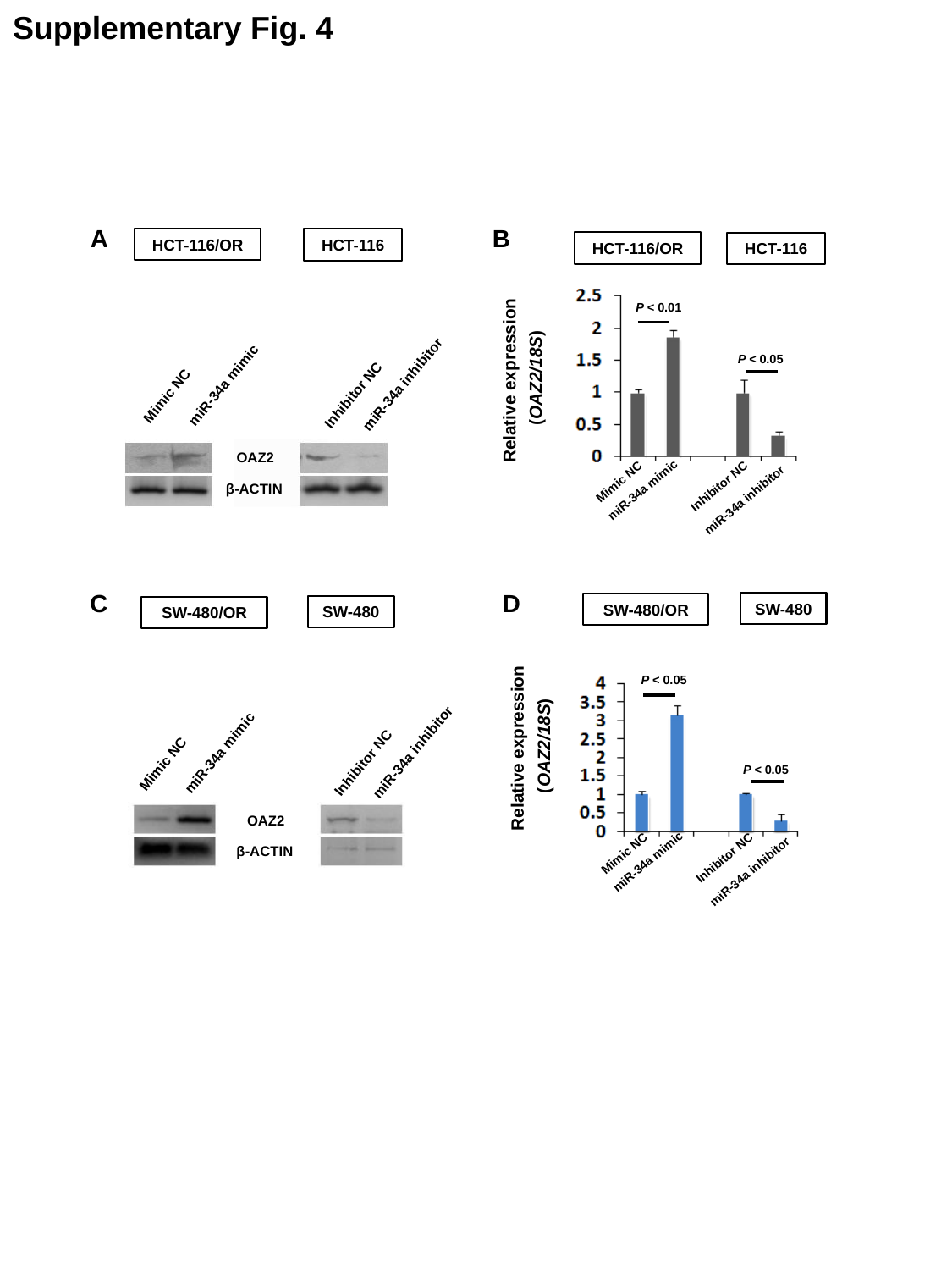

Supplementary Fig. 4
A
B
HCT-116/OR
HCT-116
HCT-116/OR
HCT-116
P < 0.01
P < 0.05
Relative expression
(OAZ2/18S)
miR-34a inhibitor
Mimic NC
miR-34a mimic
Inhibitor NC
OAZ2
Mimic NC
β-ACTIN
Inhibitor NC
miR-34a mimic
miR-34a inhibitor
C
D
SW-480
SW-480/OR
SW-480
SW-480/OR
P < 0.05
Relative expression
(OAZ2/18S)
miR-34a inhibitor
Mimic NC
miR-34a mimic
Inhibitor NC
P < 0.05
OAZ2
β-ACTIN
Mimic NC
Inhibitor NC
miR-34a mimic
miR-34a inhibitor
